# Neuroimaging phenotype characterization of early aggressive and late severe multiple sclerosis: a case-control study

**DOI:** 10.1093/braincomms/fcaf254

**Published:** 2025-06-24

**Authors:** Robert Zivadinov, Niels Bergsland, Alexander Bartnik, Dejan Jakimovski, Ferdinand Schweser, Svetlana P Eckert, David Hojnacki, Murali Ramanathan, Ralph H B Benedict, Bianca Weinstock-Guttman, Michael G Dwyer

**Affiliations:** Buffalo Neuroimaging Analysis Center, Department of Neurology, Jacobs School of Medicine and Biomedical Sciences, University at Buffalo, State University of New York, Buffalo, NY 14203, USA; Center for Biomedical Imaging at the Clinical Translational Science Institute, University at Buffalo, State University of New York, Buffalo, NY 14203, USA; Buffalo Neuroimaging Analysis Center, Department of Neurology, Jacobs School of Medicine and Biomedical Sciences, University at Buffalo, State University of New York, Buffalo, NY 14203, USA; Buffalo Neuroimaging Analysis Center, Department of Neurology, Jacobs School of Medicine and Biomedical Sciences, University at Buffalo, State University of New York, Buffalo, NY 14203, USA; Buffalo Neuroimaging Analysis Center, Department of Neurology, Jacobs School of Medicine and Biomedical Sciences, University at Buffalo, State University of New York, Buffalo, NY 14203, USA; Buffalo Neuroimaging Analysis Center, Department of Neurology, Jacobs School of Medicine and Biomedical Sciences, University at Buffalo, State University of New York, Buffalo, NY 14203, USA; Center for Biomedical Imaging at the Clinical Translational Science Institute, University at Buffalo, State University of New York, Buffalo, NY 14203, USA; Department of Neurology, Jacobs Comprehensive Multiple Sclerosis Treatment and Research Center, Jacobs School of Medicine and Biomedical Sciences, University at Buffalo, State University of New York, Buffalo, NY 14203, USA; Department of Neurology, Jacobs Comprehensive Multiple Sclerosis Treatment and Research Center, Jacobs School of Medicine and Biomedical Sciences, University at Buffalo, State University of New York, Buffalo, NY 14203, USA; Department of Pharmaceutical Sciences, University at Buffalo, State University of New York, Buffalo, NY 14203, USA; Department of Neurology, Jacobs Comprehensive Multiple Sclerosis Treatment and Research Center, Jacobs School of Medicine and Biomedical Sciences, University at Buffalo, State University of New York, Buffalo, NY 14203, USA; Department of Neurology, Jacobs Comprehensive Multiple Sclerosis Treatment and Research Center, Jacobs School of Medicine and Biomedical Sciences, University at Buffalo, State University of New York, Buffalo, NY 14203, USA; Buffalo Neuroimaging Analysis Center, Department of Neurology, Jacobs School of Medicine and Biomedical Sciences, University at Buffalo, State University of New York, Buffalo, NY 14203, USA; Center for Biomedical Imaging at the Clinical Translational Science Institute, University at Buffalo, State University of New York, Buffalo, NY 14203, USA

**Keywords:** multiple sclerosis, early aggressive, late severe, grey matter atrophy, cortical lesions

## Abstract

A subgroup of multiple sclerosis patients experiences a severe form of the disease. Although there is a lack of consensus regarding the definition of severe multiple sclerosis, it can follow two distinct temporal disability trajectories. Early aggressive multiple sclerosis is characterized by reaching an Expanded Disability Status Scale score of ≥6.0 in less than 10 years from disease onset or before reaching 40 years of age, while the same disability is reached in late severe multiple sclerosis patients over more than 10 years from onset or after the age of 40. We investigated neuroimaging phenotypes in aggressive/severe multiple sclerosis. From a retrospective, 16-year longitudinal Biomarker Understanding for Future Findings and Advancements for Long-term Outcomes in Multiple Sclerosis database, out of 3863 multiple sclerosis patients, 86 early aggressive and 368 late severe multiple sclerosis patients were included. Propensity matching for age, sex, race, disease duration, MRI scanner type and imaging protocol was used to identify early non-aggressive matched (*n* = 86) or late non-severe matched (*n* = 368) multiple sclerosis controls. Additionally, out of 675 neurologically healthy individuals from the same database, using similar matching criteria, neurologically healthy individuals were matched to early (*n* = 163) and late (*n* = 89) multiple sclerosis. MRI lesion (white matter, cortical, cervical spinal cord, thalamic dysconnectivity) and brain (whole brain, grey matter, cortex, thalamus) volumes and spinal cord area (C1-C3) measures were obtained. Statistical analyses explored group comparisons and associations with the severity of disability (Expanded Disability Status Scale defined) in aggressive/severe groups. Multiple sclerosis patients showed significantly worse MRI outcomes in all explored measures, compared to neurologically healthy individuals. Higher T2 hyperintense lesion burden (*P* = 0.008) and thalamic atrophy (*P* = 0.011) were the only significant differentiators between early aggressive and non-aggressive multiple sclerosis, while higher cortical lesion burden was the only significant correlate of disability (*P* = 0.004) in early aggressive multiple sclerosis. The late severe multiple sclerosis patients were differentiated from their matched multiple sclerosis controls by spinal cord atrophy (*P* < 0.001) and cortical atrophy (*P* < 0.001), while disability was significantly associated with spinal cord atrophy (*P* < 0.001), higher cortical lesion burden (*P* = 0.006) and thalamic atrophy (*P* = 0.024) in late severe multiple sclerosis. Early and late forms of severe multiple sclerosis share common grey matter-based pathology, with lesion burden, cortical and thalamic changes central to early aggressive multiple sclerosis and spinal cord, cortical and thalamic changes linked to late severe multiple sclerosis.

## Teaser

Zivadinov *et al.* characterized neuroimaging phenotypes of early aggressive and late severe multiple sclerosis, highlighting the critical role of cortical lesions, white matter lesion burden and thalamic pathology in early aggressive multiple sclerosis and spinal cord, cortical, thalamic and global pathology in late severe multiple sclerosis.

## Graphical Abstract

**Graphical Abstract:**
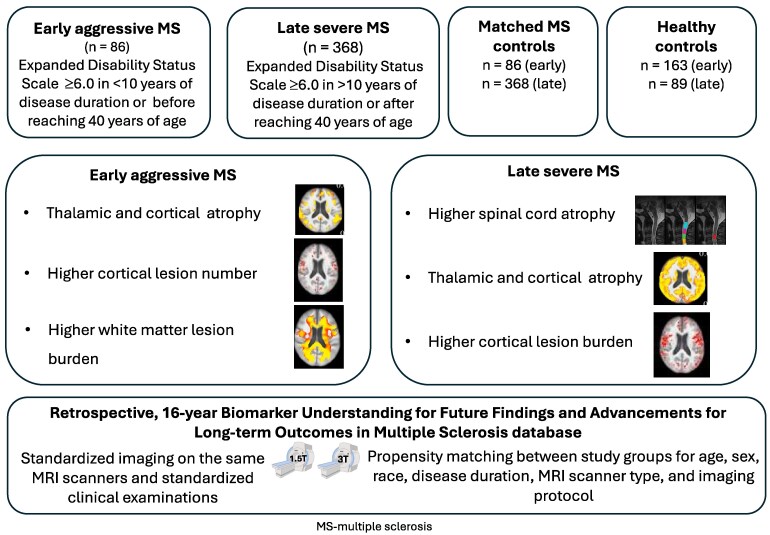

## Introduction

Multiple sclerosis is a chronic inflammatory demyelinating disease of the central nervous system (CNS) that can lead to substantial disability, manifesting with diverse clinical patterns and outcomes that differ from person to person and may change over time.^1-3^ The trajectory of multiple sclerosis is highly individual, with severity levels ranging from mild to severe.^2^ Typically, most multiple sclerosis patients will accumulate disability and some will transition into a progressive phase over time.^1-3^

A subtype of multiple sclerosis has long been identified as having what could be described as a severe multiple sclerosis.^4-7^ This form of the disease can be evident from the onset or emerge later during its progression and can follow two distinct temporal disability trajectories. Individuals with early aggressive multiple sclerosis reach an Expanded Disability Status Scale (EDSS) score of ≥6.0 within less than 10 years from disease onset^8-11^ or before reaching 40 years of age,^9,12^ while those with late severe multiple sclerosis reach the same disability progression over more than 10 years from onset or after age of 40.^6,7^ An international forum provided a comprehensive review of available information on the early aggressive multiple sclerosis, but failed to come to consensus on a unique definition.^4,5^ Such patients often experience rapid and severe disability within a relatively short time from disease onset. Late severe multiple sclerosis is observed in the subpopulation of multiple sclerosis patients in which progression to severe disability occurs over an extended period, with ageing-related processes believed to play a modulating role.^1,3,6,7^ The prevalence of early aggressive multiple sclerosis has not been established, but it may vary between 5 and 10% of patients,^4,8,12^ while the figures are somewhat higher for late severe multiple sclerosis.^4,6^

Previous research has determined that clinical and demographic indicators of early aggressive multiple sclerosis may include male sex,^13^ older age at onset,^9,13,14^ severe relapses with multifocal presentation,^14^ incomplete remission,^14^ involvement of motor, cognitive, cerebellar or sphincteric functions,^14^ presence of pyramidal signs in the first year of disease evolution,^9^ frequent relapses in the first years after onset,^15^ short inter-attack interval^16^ and early accrual of disability with superimposed attacks.^9,16^ MRI features of early aggressive multiple sclerosis may include ≥20 T2 lesions or ≥2 contrast-enhancing lesions at the time of disease onset,^10^ higher number of T1 hypointense black holes early in the disease,^17^ presence of infratentorial lesions,^18^ spinal cord (SC) lesions and/or atrophy,^19^ cortical and deep grey matter (GM) atrophy,^20^ thalamic atrophy,^21^ early presence of cortical^22^ and chronic active lesions.^22-24^ A recent study showed that late severe multiple sclerosis is characterized by extensive GM pathology, predominantly located in the cortex and thalamus.^6^

To date, no studies have characterized the neuroimaging phenotype of early aggressive versus late severe multiple sclerosis within population-based cohorts and these disease forms have been rarely studied retrospectively or prospectively. This is in part because there is a lack of consensus on the exact definition of severe multiple sclerosis and its management and treatment guidelines.^4,5^ Most of the neuropathological studies researched late severe multiple sclerosis.^25^ The primary question to address is whether early aggressive multiple sclerosis represents a distinct disease phenotype driven by fundamentally different mechanisms of progression compared to late severe multiple sclerosis, or if it merely reflects an intensified stage along a continuum of the same underlying pathology.^4^ It remains to be clarified whether the variability in the development of early aggressive versus late severe multiple sclerosis is driven by distinct pathological factors—such as GM, white matter (WM) or SC involvement—or if a shared underlying mechanism governs the course of disease progression.

The objective of this study was to characterize neuroimaging phenotypes of early aggressive versus late severe multiple sclerosis. Based on different temporal disability trajectories of these patient groups (lower age and disease duration in early aggressive versus late severe multiple sclerosis), we also included early and late matched multiple sclerosis controls and neurologically healthy individuals (NHI) for comparison purposes.

## Materials and methods

### Study population

**Figure 1:**
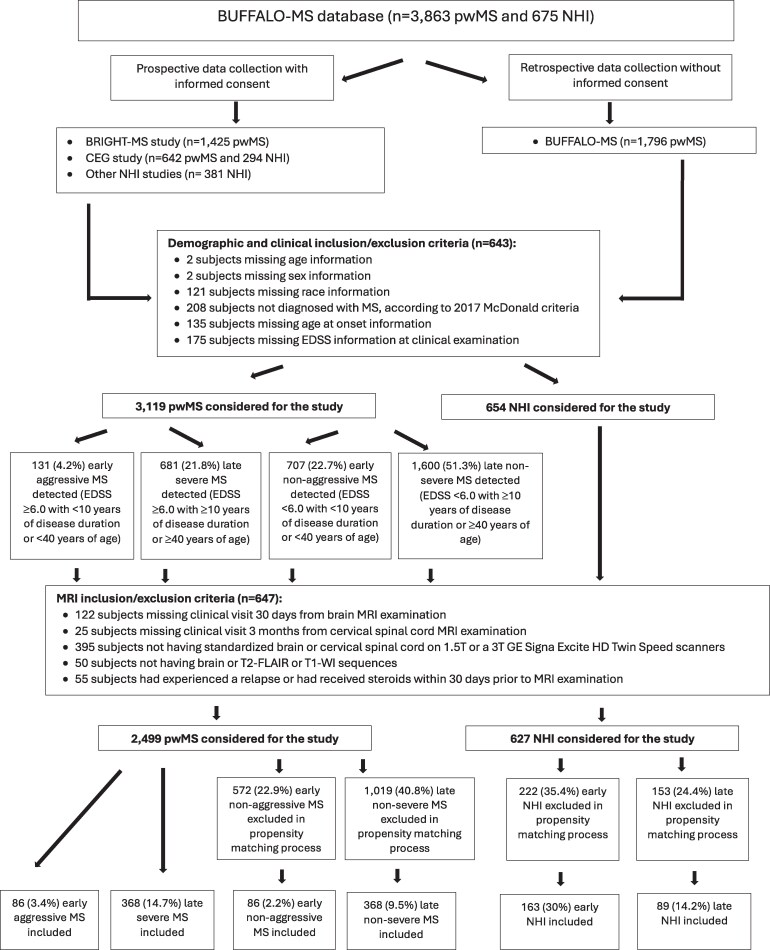
**Inclusion and exclusion criteria for the aggressive and late severe people with multiple sclerosis from BUFFALO-MS database.** CEG: Cardiovascular Environmental and Genetic study.

This is a retrospective, case-control study from subjects included in the longitudinal Biomarker Understanding for Future Findings and Advancements for Long-term Outcomes in Multiple Sclerosis (BUFFALO-MS) database.^24,26,27^ The BUFFALO-MS database (Fig. 1) is a retrospective database of multiple sclerosis patients and NHI collected over a period of 16 years (2007-2024). The database contains data on 3863 multiple sclerosis patients and 675 NHI collected prospectively (BRIGHT-MS, Cardiovascular Environmental and Genetic study and other studies in NHI),^24,26,27^ or retrospectively (multiple sclerosis only) over the same time period. All multiple sclerosis patients were followed at the Jacobs Comprehensive Multiple Sclerosis Treatment and Research Center, University at Buffalo, Buffalo, NY, while the NHI were enrolled at the Buffalo Neuroimaging Analysis Center, University at Buffalo, Buffalo, NY. The retrospective collection of demographic, clinical and MRI assessments occurred on a bi-annual basis and was cross-referenced to electronic medical records by trained coordinators and medical doctors.^26,27^ Disease status and severity were evaluated by multiple sclerosis-trained clinicians using standardized physical and neurological evaluations, including EDSS. There are over 35 000 clinical visits, 40 000 brain MRI scans and 15 000 cervical SC MRI scans available in the database. NHI include non-familial relatives of patients with multiple sclerosis, as well as participants recruited through local advertisements and the Buffalo Research Registry, which includes approximately 7100 subjects, representative of the community’s diversity. NHI were enrolled if they presented with normal neurological and MRI findings within clinical expectations for their age.

Figure 1 shows a flow chart of the inclusion and exclusion criteria for the subjects selected for this study. The study inclusion criteria were: (i) diagnosed with multiple sclerosis based on the 2017-revised McDonald criteria (all subjects in the BUFFALO-MS database were retrospectively classified as fulfilling or not fulfilling 2017-revised McDonald criteria in 2024),^28^ (ii) having relapsing or progressive multiple sclerosis, according to the 2013 Lublin criteria,^2^ (iii) availability of demographic (age, sex, race) and clinical (age at onset, disease duration, EDSS) data at the time of MRI examination, (iv) having a brain MRI with available T2-fluid-attenuated inversion recovery (FLAIR), T1-weighted imaging (WI) and T2-WI, obtained no longer than 30 days from a clinical examination, (v) having early aggressive multiple sclerosis, defined as reaching ≥6.0 EDSS score (confirmed after at least 6 months from the initial EDSS score determination) within less than 10 years from disease onset or before reaching 40 years of age, (vi) having late severe multiple sclerosis, defined as reaching ≥6.0 EDSS confirmed score after 10 years from disease onset or after age of 40, (vii) being an age, sex, race, disease duration, MRI scanner type and protocol matched multiple sclerosis control to early aggressive or late severe multiple sclerosis and (viii) being an age, sex, race, MRI scanner type and protocol matched as early or late NHI. We also included a cervical SC MRI, when available for multiple sclerosis patients, that was obtained no longer than 3 months from a clinical and brain MRI examination. The exclusion criteria were: (i) experienced a relapse or received steroids within 30 days prior to MRI examination, (ii) women who were pregnant or lactating and (iii) pre-existing medical conditions known to be associated with brain pathology (cerebrovascular disease, positive history of alcohol abuse).

To control for potential confounding variables, propensity score matching was performed using demographic, clinical and MRI characteristics, including age, sex, race, disease duration, MRI scanner type and imaging protocol. This approach identified two matched comparison groups from the BUFFALO-MS database: early non-aggressive multiple sclerosis controls (*n* = 86) and late non-severe multiple sclerosis controls (*n* = 368), each matched to the respective early aggressive (*n* = 86) and late severe (*n* = 368) multiple sclerosis groups. In addition, from a pool of 675 NHI within the same database, matched controls were selected for early aggressive (*n* = 163) and late severe (*n* = 89) multiple sclerosis patients using the same criteria. Written informed consent was waived for this study for multiple sclerosis patients due to the retrospective nature, while all NHI had provided informed consent as part of other studies, although their data were used here retrospectively. The study was approved by the Institutional Review Board of the University at Buffalo.

### Demographic and clinical data

Demographic and clinical data were collected from the BUFFALO-MS database, as described above and shown in Tables 1 and 2. Additional data on cardiovascular risk factors including smoking, obesity, hypertension, hyperlipidaemia, heart disease and obesity were available from the same database.

**Table 1:** Demographic, clinical and MRI acquisition characteristics of the early aggressive, non-aggressive and control populations

	Early aggressive multiple sclerosis (*n* = 86)	Early non-aggressive multiple sclerosis (*n* = 86)	Early NHI (*n* = 163)	*P*-value
Female, *n* (%)	53 (61.6)	53 (61.6)	101 (61.9)	0.998
Race, *n* (%)				
Caucasian	66 (74.4)	66 (74.4)	122 (75.3)	0.996
African-American	22 (25.6)	22 (25.6)	41 (25.2)	
Disease course, *n* (%)				
Relapsing-remitting	47 (54.7)	70 (81.4)	NA	<0.001
Secondary progressive	27 (31.4)	10 (11.6)		
Primary progressive	12 (14)	6 (7)		
Age in years, mean (SD)	47.4 (11.4)	47.4 (11.3)	47.8 (16.6)	0.948
Age at onset in years, mean (SD)	38.3 (14.2)	38.3 (13.8)	NA	0.983
BMI, mean (SD)	27.9 (8.7)	28.5 (5.6)	27.1 (6.3)	0.717
Education, years, mean (SD)	13.6 (2.4)	14.1 (2.4)	14 (2.3)	0.299
Disease duration (years), mean (SD)	8.5 (5.3)	8.6 (5.3)	NA	0.908
EDSS, median (IQR)	6.0 (6.0-6.5)	2.5 (1.5-3.5)	NA	<0.001
Brain MRI (*n*, %)				
1.5 T	38 (44.2)	38 (44.2)	72 (44.2)	0.999
3 T	48 (55.8)	48 (55.8)	91 (55.8)	
Cervical spinal cord MRI (*n*, %)				
1.5 T	29 (33.7)	27 (31.4)	NA	0.488
3 T	35 (40.7)	42 (48.8)		
Missing	22 (25.6)	17 (19.8)		
DMT, *n* (%)				
Interferon-beta	24 (27.9)	31 (19.0)	NA	<0.001
Glatiramer acetate	19 (22.1)	9 (9.5)		
Oral therapies	10 (11.6)	15 (2.4)		
Anti-CD20	8 (9.3)	0 (7.1)		
Natalizumab	10 (11.6)	15 (0.0)		
Other	3 (7.1)	6 (7.0)		
No therapy	10 (11.6)	10 (11.6)		

*P*-values derived from chi-square test, Student’s *t*-test, Mann-Whitney *U*-test and ANOVA. Interferon-beta therapies include: intramuscular and subcutaneous interferon-beta 1a and subcutaneous interferon-beta 1b. Glatiramer acetate includes Copaxone®. Oral therapies include teriflunomide, dimethyl-fumarate, fingolimod and diroximel fumarate. Anti-CD-20 therapies include ocrelizumab. Other therapies include mitoxantrone and methylprednisolone.

BMI, body mass index; SD, standard deviation; DMT, disease-modifying treatment; NA, not available.

**Table 2:** Demographic, clinical and MRI acquisition characteristics of the late severe, non-severe and control populations

	Late severe multiple sclerosis (*n* = 368)	Late non-severe multiple sclerosis (*n* = 368)	Late NHI (*n* = 89)	*P*-value
Female, *n* (%)	278 (75.5)	279 (75.6)	66 (74.2)	0.958
Race, *n* (%)				
Caucasian	346 (94)	346 (94)	84 (94.4)	0.868
African-American	22 (6)	22 (6)	5 (5.6)	
Disease course, *n* (%)				
Relapsing-remitting	100 (27.2)	292 (79.1)	NA	<0.001
Secondary progressive	239 (64.9)	70 (19)		
Primary progressive	29 (7.9)	7 (1.9)		
Age in years, mean (SD)	57.3 (7.7)	57.1 (7.7)	56.8 (9.1)	0.852
Age at onset in years, mean (SD)	32.6 (0.2)	32.8 (9.2)	NA	0.819
BMI, mean (SD)	27.6 (6.1)	27.4 (5.5)	26.4 (5.8)	0.792
Education, years, mean (SD)	14.7 (2.8)	15 (2.6)	14.8 (2.5)	0.165
Disease duration (years), mean (SD)	24.1 (8.8)	23.8 (8.8)	NA	0.634
EDSS, median (IQR)	6.5 (6.0-7.5)	3.0 (2.0-3.5)	NA	<0.001
Brain MRI (*n*, %)				
1.5 T	141 (38.3)	141 (38.3)	34 (38.2)	0.999
3 T	227 (61.7)	227 (61.7)	55 (61.8)	
Cervical spinal cord MRI (*n*, %)				
1.5 T	105 (28.5)	100 (27.2)	NA	0.300
3 T	177 (48.1)	202 (54.9)		
Missing	86 (23.4)	66 (17.9)		
DMT, *n* (%)				
Interferon-beta	97 (26.4)	141 (38.3)	NA	<0.001
Glatiramer acetate	65 (17.7)	54 (14.7)		
Oral therapies	19 (5.2)	30 (8.2)		
Anti-CD20	32 (8.7)	19 (5.2)		
Natalizumab	27 (7.3)	25 (6.8)		
Other	51 (13.9)	30 (8.2)		
No therapy	77 (20.9)	69 (18.8)		

*P*-values derived from chi-square test, Student’s *t*-test, Mann-Whitney *U*-test and ANOVA. Interferon-beta therapies include intramuscular and subcutaneous interferon-beta 1a, subcutaneous interferon-beta 1b and peginterferon-beta 1a. Glatiramer acetate includes Copaxone® and Glatopa®. Oral therapies include teriflunomide, dimethyl-fumarate, fingolimod, ozanimod, siponimod, cladribine and diroximel fumarate. Anti-CD-20 therapies include ocrelizumab, ofatumumab and rituximab. Other therapies include mitoxantrone, methylprednisolone, intravenous immunoglobulin, azathioprine, methotrexate and mycophenolate mofetil.

BMI, body mass index; SD, standard deviation; DMT, disease-modifying treatment; NA, not available.

### MRI acquisition

The MRI exams used in the present study were obtained between 2007 and 2023 on either a 1.5T or a 3T GE Signa Excite HD Twin Speed scanner using head and neck coils (GE, Milwaukee, Wisconsin, USA). Neither scanner underwent major hardware changes over the study period. The software on both scanners was upgraded in November 2017. Acquisition parameters are presented in Supplementary Table 1.

### MRI analyses

All image analyses were performed in a blinded manner without knowledge of the subjects’ disease status, demographic details or clinical conditions.

#### Lesion analysis

T2 lesion volume (LV), T1-LV and contrast enhancing-LV and lesion number (LN) were obtained using a semi-automated contouring/thresholding technique using Java Image Manipulation version 6.0.^29^

**Figure 2:**
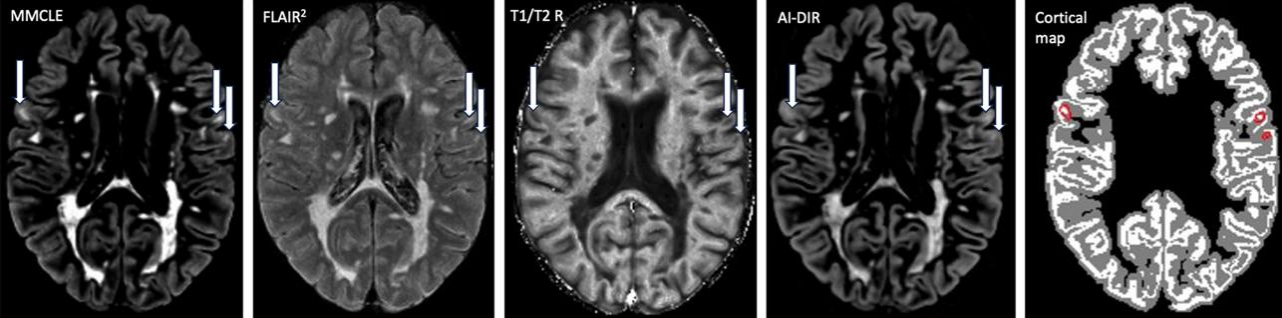
**Representative example of the images and layout used for simultaneous cortical lesion detection and/or review.** Examples of well-visualized cortical lesions are highlighted (arrows). From left to right: multi-modal cortical lesion-enhanced image, a combination of FLAIR square (FLAIR2) and AI-double inversion recovery (AI-DIR); FLAIR2, a combination of FLAIR and T2-WI; T1/T2 ratio (T1/T2 R) image; AI-DIR, synthesized based on a deep neural network from the FLAIR, T1, proton density and T2 images and cortical map from FastSurfer (actual cortex shown in white, cortical lesions shown in red circles, voxels within 2 mm of cortex, including from above and below, in grey).

Cortical LN/LV detection was conducted via a combination of previously described and validated retrospective methods, including FLAIR2,^30^ artificial intelligence (AI)-double inversion recovery^31^ and T1/T2 ratio.^32^ The following raw images were used: T2-WI, 3D T1-WI (pre-contrast) and T2-FLAIR. Raw images were pre-processed to remove bias field inhomogeneities,^33^ aligned to Montreal Neurological Institute space,^34^ upsampled to 1-mm isotropic voxels, intensity standardized and de-skulled.^35^ FLAIR2 images were created via multiplication of aligned T2-FLAIR and T2-WI,^30^ and T1/T2 ratio I images were created by dividing T1-WI by the T2-WI.^32^ AI-double inversion recovery images were created via a trained deep generative neural network as described by Bouman *et al*.^31^ To provide additional context during lesion delineation, FastSurfer^36^ was used to create cortical surface maps. To further facilitate manual review and improve contrast, multi-modal cortical lesion-enhanced images were created via voxel-wise multiplication of AI-double inversion recovery and FLAIR2 images.^37^ A trained in-house semantic segmentation deep neural network was used as a preliminary step in lesion delineation to improve sensitivity and reliability. Lesions were identified by the expert neuroimagers in line with pre-defined criteria using a custom 3D Slicer module, based on simultaneous reference to all four lesion-sensitive contrasts (Fig. 2).^38^

Lesion-based thalamic dysconnectivity, representing structural dysconnectivity of the thalamus, was assessed using the Network Modification tool.^39,40^

T2-STIR images of the upper SC were first pre-processed using the Synthetic Multi-Orientation Resolution Enhancement technique.^41^ Use of Synthetic Multi-Orientation Resolution Enhancement has been shown to improve automated segmentation of the upper cervical SC.^42^ Internal testing showed that it helped facilitate SC lesion quantification as well. Next, the Spinal Cord Toolbox^43^ was used for quantifying SC-LN/LV, via the *sct_deepseg_lesion* programme.^44^

#### Brain volumes and SC cross-sectional area analysis

Whole brain volume, GM volume (GMV), WM volume, cortical volume and lateral ventricles volume (LVV) were determined on 3D T1-WI using cross-sectional Structural Image Evaluation using Normalisation of Atrophy.^45^ Lesion filling was performed prior to segmentation to minimize tissue misclassification.^34^ Similarly, thalamic volume (TV) was also calculated on 3D T1-WI using FMRIB’s Integrated Registration and Segmentation Tool.^46^

Mean upper cervical cord area (MUCCA) at C1-C3 was assessed on T2-STIR images using *sct_deepseg_sc* programme.^44^

#### Voxel-wise analysis

A custom template was first created with the antsMultivariateTemplateConstruction2.sh script from Advanced Normalization Tools using the lesion-filled brain images from all multiple sclerosis patients. Each lesion-filled brain image was then non-linearly registered to the template using Advanced Normalization Tools. Lesion probability mapping (LPM) of T2-LV, T1-LV and cortical LV was performed as previously described.^47^ Similarly, voxel-based morphometry (VBM) was conducted using the non-linearly registered and modulated GM partial volume estimates obtained from cross-sectional Structural Image Evaluation using Normalisation of Atrophy. For both LPM and VBM, voxel-wise general linear models were tested with the Permutation Analysis of Linear Models tool^48^ using tail acceleration (500 permutations),^49^ adjusting for age and sex. Threshold-free cluster enhancement was used and false discovery rate-corrected *P* < 0.05 was considered significant.^50^

### Statistical analyses

All statistical analyses and visualizations were performed using SPSS version 29.0 (IBM, Armonk, NY, USA) and R 4.4.2 software. Data distributions were assessed using visual inspection of the data and histograms, and model assumptions were checked using *Q*-*Q* plots. Categorical variables were compared using chi-square tests. Group comparisons were performed parametrically using Student’s *t*-test (for normally distributed data) and non-parametrically using Mann-Whitney *U*-test.

Analysis of covariance adjusted for efficacy of disease-modifying treatment, MRI scanner type and protocol was performed between early aggressive and late severe multiple sclerosis and their matched multiple sclerosis controls, and between early and late matched multiple sclerosis controls and the NHI. For the purpose of the data analyses, the disease-modifying treatment efficacy was categorized as none, low/moderate (interferon-beta, glatiramer acetate oral and immunosuppressive therapies) and high efficacy (monoclonal antibodies). Cohen’s *d* effect size was calculated.

A stepwise logistic regression model was used to identify the factors that best discriminate between early aggressive and late severe multiple sclerosis and their matched multiple sclerosis controls, using brain and SC MRI measures that were found to be significantly different in group comparison analyses. Additionally, linear stepwise regression analyses were used to determine the relationship between clinical disability (EDSS) with the brain and SC MRI measures in early aggressive and late severe multiple sclerosis study groups. The logistic and linear regression models included age, disease duration, disease-modifying treatment efficacy (none, low/moderate and high) and MRI scanner type and protocol as covariates. To facilitate interpretation of the models, *z*-scored MRI variables were used as inputs to the regression analyses.

*P*-values lower than 0.05 were considered statistically significant, and *P* < 0.1 a statistical trend. The Benjamini-Hochberg procedure was used to correct for multiple comparisons by controlling the false discovery rate and only corrected *P* values are shown.

## Results

### Demographic and clinical characteristics of the study populations

Figure 1 shows that out of 3863 multiple sclerosis patients and 675 NHI in the BUFFALO-MS database, 3119 multiple sclerosis patients and 654 NHI were considered for the study, based on inclusion/exclusion demographic and clinical criteria that were missing for 643 subjects. Of those, 131 (4.2%) presented with early aggressive multiple sclerosis and 681 (21.8%) presented with late severe multiple sclerosis, while the figures were 707 (22.8%) for early non-aggressive and 1600 (51.3%) for late non-severe multiple sclerosis. Additionally, 647 subjects were excluded due to MRI inclusion/exclusion criteria for the study. Of the 2499 multiple sclerosis patients and 627 NHI fulfilling all inclusion/exclusion criteria for the study, 86 early aggressive and 368 late severe multiple sclerosis were identified (Fig. 1 and Tables 1 and 2). The rest of the subjects entered the propensity matching process, and 86 early non-aggressive and 368 late non-severe matched multiple sclerosis controls and 163 early and 89 late NHI were selected, respectively (Fig. 1 and Tables 1 and 2). The proportion of subjects selected for the study groups did not differ significantly (*P* = 0.886) across the data collection period (2007-2023).

Tables 1 and 2 provide demographic, clinical and MRI acquisition characteristics of the study population. The mean age in early aggressive multiple sclerosis was 47.4 years, with 8.5 years of disease duration, and the median EDSS was 6.0 (IQR 6.0-6.5), while the figures were 57.3 and 24.1 years, and 6.5 (IQR 6.0-7.5) in late severe multiple sclerosis, respectively. The early aggressive and late severe multiple sclerosis were well matched to their multiple sclerosis controls and NHI, for age, age at onset, sex, race, BMI, education and MRI scanner type (1.5T or 3T) and MRI brain and cervical SC protocol. Cervical SC MRI was present in 74.4% of early aggressive and 76.6% of late severe multiple sclerosis. As expected, both early aggressive and late severe multiple sclerosis had significantly higher EDSS and more progressive disease courses compared to their matched multiple sclerosis controls. There was also significantly higher use of monoclonal antibodies for individuals with early aggressive and late severe multiple sclerosis.

There were no significant differences in cardiovascular risk factors between those with early aggressive and late severe multiple sclerosis and their matched multiple sclerosis controls and NHI (data not shown).

When early aggressive were compared to late severe multiple sclerosis, a significantly higher frequency of African-Americans (25.6% versus 6%, *P* < 0.001) and males (38.4% versus 24.5%, *P* = 0.011), higher age at onset (38.3 versus 32.6 years, *P* < 0.001) and significantly lower frequency of progressive multiple sclerosis (45.3% versus 72.8%, *P* < 0.001) were observed.

### MRI lesion differences between the study populations

**Figure 3:**
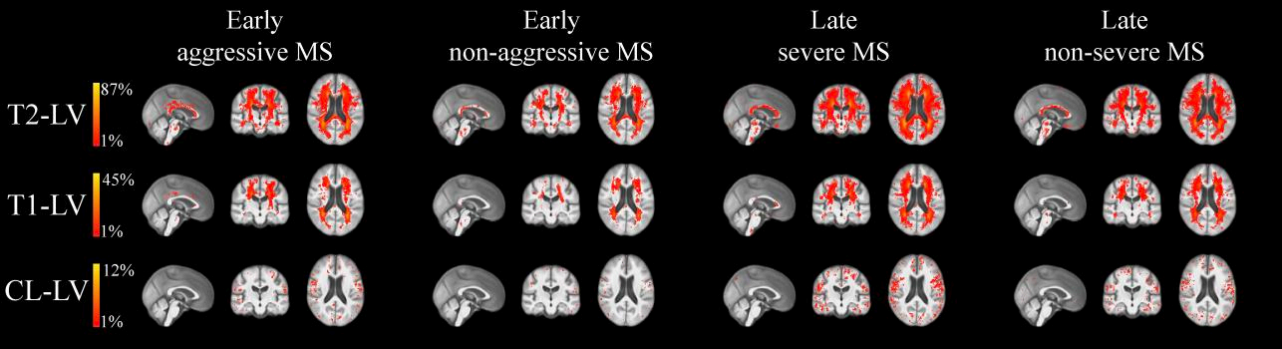
**LPMs of T2-LV, T1-LV and cortical LV.** Colours represent the percentage of individuals presenting with a lesion. LPMs are overlaid on the study-specific template in which the mid-sagittal, -coronal and -axial slices are shown. Early aggressive multiple sclerosis and early non-aggressive multiple sclerosis groups had 86 individuals in each group. Late severe multiple sclerosis and late non-severe multiple sclerosis groups had 368 individuals in each group.

**Figure 4:**
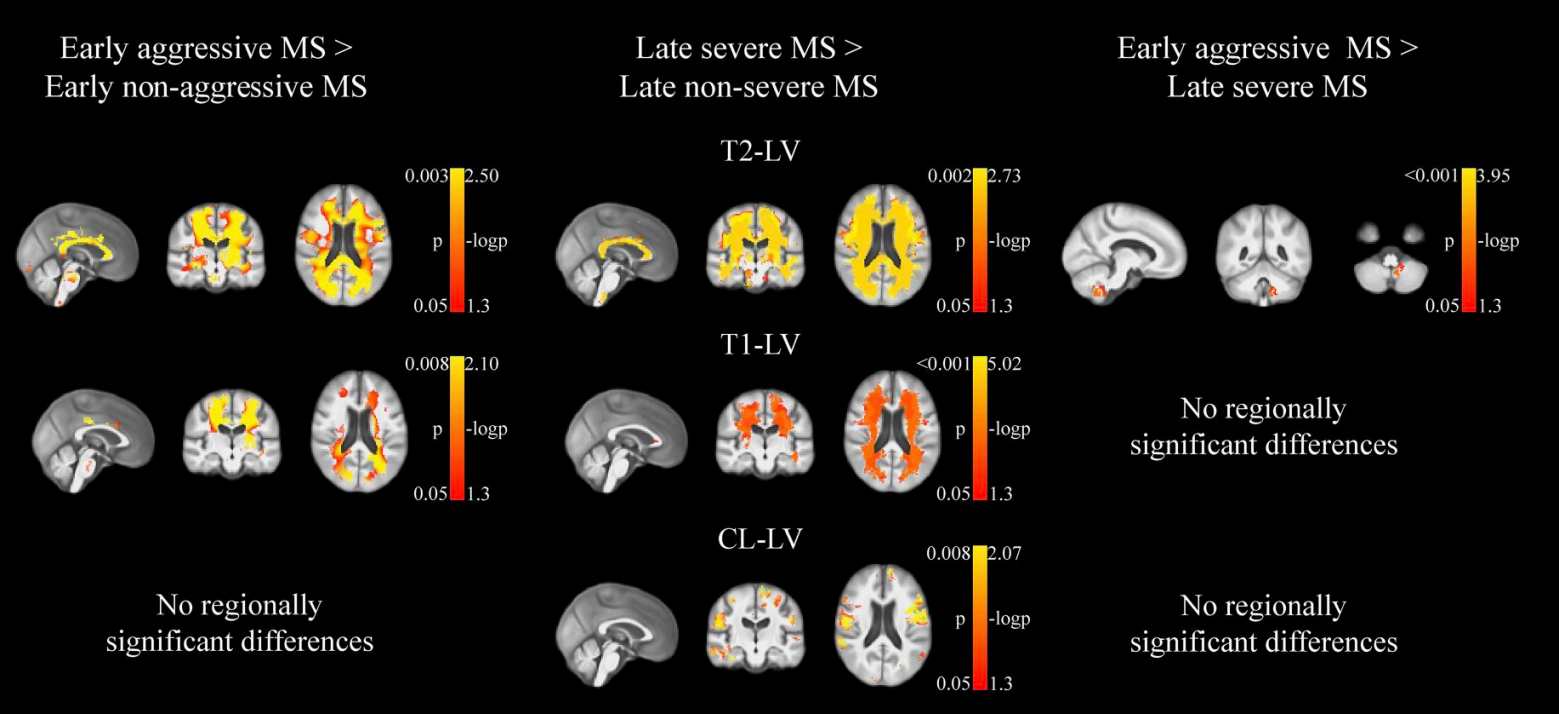
**Statistical analyses of LPMs for T2-LV, T1-LV and cortical LV.** Permutation testing with threshold-free cluster enhancement was used while correcting for age and sex. False discovery rate *P* < 0.05 voxels are shown. LPMs are overlaid on the study-specific template in which the mid-sagittal, -coronal and -axial slices are shown, except for the ‘early aggressive multiple sclerosis > late severe multiple sclerosis’ comparison, where the cerebellum is shown. Early aggressive multiple sclerosis and early non-aggressive multiple sclerosis groups had 86 individuals in each group. Late severe multiple sclerosis and late non-severe multiple sclerosis groups had 368 individuals in each group.

Tables 3 and 4 show lesion characteristics of the study groups. Data for contrast-enhancing, cortical and SC lesions were not available for NHI. LPMs are shown in Fig. 3, while statistical comparisons are shown in Fig. 4.

**Table 3:** MRI outcome comparisons of the early aggressive, non-aggressive and control populations

	Early aggressive multiple sclerosis (*n* = 86)	Early non-aggressive multiple sclerosis (*n* = 86)	Early NHI (*n* = 163)	*P*-value*	Cohen’s d*	*P*-value**
Lesion MRI outcomes						
T2-LV	18.8 (18)	10.2 (10.6)	1.6 (3)	<0.001	0.578	<0.001
T1-LV	5.3 (6.7)	2.5 (3.5)	0.9 (0.6)	0.004	0.800	<0.001
CE-LN	2.4 (4.2)	1.4 (3.3)	NA	0.094	0.267	NA
CE-LV	0.2 (0.9)	0.1 (0.4)	NA	0.227	0.186	NA
CL-LN	19.7 (21.3)	11.5 (13.6)	NA	0.004	0.459	NA
CL-LV	0.7 (1.1)	0.4 (0.7)	NA	0.02	0.362	NA
TDSC	5.3 (3.1)	3.4 (3.1)	0.3 (0.5)	0.002	0.447	<0.001
SC-LN	5.4 (3)	4.9 (3.3)	NA	0.514	0.185	NA
SC-LV	0.3 (0.3)	0.2 (0.2)	NA	0.323	0.246	NA
Brain volume and spinal cord area MRI outcomes						
WBV	1453.8 (94.8)	1481.2 (108.7)	1542.3 (92.3)	0.077	0.268	<0.001
GMV	694.7 (84.9)	718.4 (89.2)	754.5 (59.9)	0.077	0.273	<0.001
WMV	759.2 (87.0)	762.8 (97.7)	787.8 (51.8)	0.778	0.039	<0.001
CV	547.9 (71.5)	569.3 (73.1)	612.8 (53.2)	0.035	0.296	0.01
LVV	57.2 (22.8)	56.9 (21.9)	34.8 (15.8)	0.013	0.373	<0.001
TV	13.4 (2.2)	14.7 (2.3)	15 (1.4)	<0.001	0.561	<0.001
MUCCA	56.1 (10.9)	58.9 (12)	NA	0.160	0.271	NA

All measures are reported as mean (standard deviation). Volumes are reported in millilitres. Thalamic dysconnectivity is a unitless measure. MUCCA is reported in mm^2^. *P*-values derived from analysis of covariance, corrected for efficacy of disease-modifying treatment, MRI scanner field strength and protocol. False discovery rate-corrected *P*-values and Cohen’s *d* effect size are shown between early aggressive versus early non-aggressive matched multiple sclerosis controls.

CL, cortical; TDSC, thalamic dysconnectivity; WBV, whole brain volume; WMV, white matter volume; CV, cortical volume; NA, not available.

^*^Early aggressive versus early non-aggressive matched multiple sclerosis controls.

^**^Early non-aggressive matched multiple sclerosis controls versus early NHI.

**Table 4:** MRI outcome comparisons in the late severe, non-severe and control populations

	Late severe multiple sclerosis (*n* = 368)	Late non-severe multiple sclerosis (*n* = 368)	Late NHI (*n* = 89)	*P*-value*	Cohen’s d*	*P*-value**
Lesion MRI outcomes						
T2-LV	20.6 (18.4)	12 (11.5)	2.4 (6.8)	<0.001	0.565	<0.001
T1-LV	5.4 (6.4)	3.0 (4.0)	0.5 (2.7)	<0.001	0.754	<0.001
CE-LN	1.5 (2.6)	1.2 (2.1)	NA	0.066	0.150	NA
CE-LV	0.1 (0.3)	0.1 (0.1)	NA	0.014	0.208	NA
CL-LN	16.8 (17.7)	11.2 (14.5)	NA	<0.001	0.351	NA
CL-LV	0.6 (0.8)	0.4 (0.7)	NA	<0.001	0.352	NA
TDSC	4.8 (4.1)	3.6 (3)	0.4 (0.9)	<0.001	0.335	<0.001
SC-LN	5.1 (3.4)	4.4 (2.9)	NA	0.022	0.228	NA
SC-LV	0.2 (0.2)	0.18 (0.2)	NA	0.042	0.199	NA
Brain volume and spinal cord area MRI outcomes						
WBV	1409.3 (113.9)	1444.9 (93.6)	1518.6 (91.8)	<0.001	0.383	<0.001
GMV	677.9 (92.8)	722.3 (74.6)	761.2 (70.7)	<0.001	0.535	<0.001
WMV	734.2 (108.6)	727.0 (83.3)	787.4 (75.4)	0.398	0.074	<0.001
CV	532.7 (71.7)	568.1 (56)	585.7 (52.2)	<0.001	0.551	0.008
LVV	66.7 (27.1)	54.5 (22.0)	38.7 (15.2)	<0.001	0.343	<0.001
TV	13.0 (2.5)	14.2(1.8)	15.1(1.6)	<0.001	0.494	<0.001
MUCCA	49.9(11.2)	57.8(9.7)	NA	<0.001	0.753	NA

All measures are reported as mean (standard deviation). Volumes are reported in millilitres. Thalamic dysconnectivity is a unitless measure. MUCCA is reported in mm^2^. *P*-values derived from analysis of covariance, corrected for efficacy of disease-modifying treatment, MRI scanner field strength and protocol. False discovery rate-corrected *P*-values and Cohen’s *d* effect size are shown between late severe versus late non-severe matched multiple sclerosis controls.

CL, cortical; TDSC, thalamic dysconnectivity; SC, spinal cord; WBV, whole brain volume; WMV, white matter volume; CV, cortical volume; NA, not available.

^*^Late severe versus late non-severe matched multiple sclerosis controls.

^**^Late non-severe matched multiple sclerosis controls versus late NHI.

Early aggressive multiple sclerosis showed significantly higher T2-LV (*P* < 0.001), thalamic dysconnectivity (*P* = 0.002), T1-LV (*P* = 0.004), cortical LN (*P* = 0.004) and cortical LV (*P* = 0.02), compared to early non-aggressive multiple sclerosis (Table 3). In LPM analysis, early aggressive multiple sclerosis presented with increased T2-LV throughout large swaths of the WM, including the corpus callosum, deep WM, subcortical WM and portions of the brainstem, while increased T1-LV probability was somewhat more restricted but still included the corticospinal tracts, periventricular WM and brainstem; no regionally significant differences were found for cortical LV. Lesion probability was not increased for any region in the early non-aggressive multiple sclerosis group. Compared to late severe multiple sclerosis, early aggressive multiple sclerosis showed increased T2-LV probability in a cluster restricted to the left cerebellum; late severe multiple sclerosis did not present with any areas of increased lesion probability compared to their early aggressive counterparts. Early non-aggressive multiple sclerosis showed significantly higher T2- and T1-LVs and thalamic dysconnectivity (all *P* < 0.001), compared to NHI.

Late severe multiple sclerosis showed significantly higher T2-LV (*P* < 0.001), T1-LV (*P* < 0.001), cortical LV (*P* < 0.001), cortical LN (*P* < 0.001), thalamic dysconnectivity (*P* < 0.001), SC-LN (*P* = 0.022) and SC-LV (*P* = 0.042), compared to late non-severe multiple sclerosis (Table 4). In LPM analysis, late severe multiple sclerosis showed a similar pattern as was evidenced in the comparison between early aggressive multiple sclerosis and its matched counterparts but was even more extensive. Specifically, the identified T2-LV cluster spanned nearly the entire WM, while T1-LV probability was slightly more restricted. In addition, increased cortical LV probability was seen throughout much of the cortex. Late non-severe multiple sclerosis did not present with any areas of increased lesion probability compared to their late severe multiple sclerosis counterparts. Late non-severe multiple sclerosis showed significantly higher T2- and T1-LVs and thalamic dysconnectivity (all *P* < 0.001), compared to NHI.

### MRI volumetric and cross-sectional area differences between the study populations

**Figure 5:**
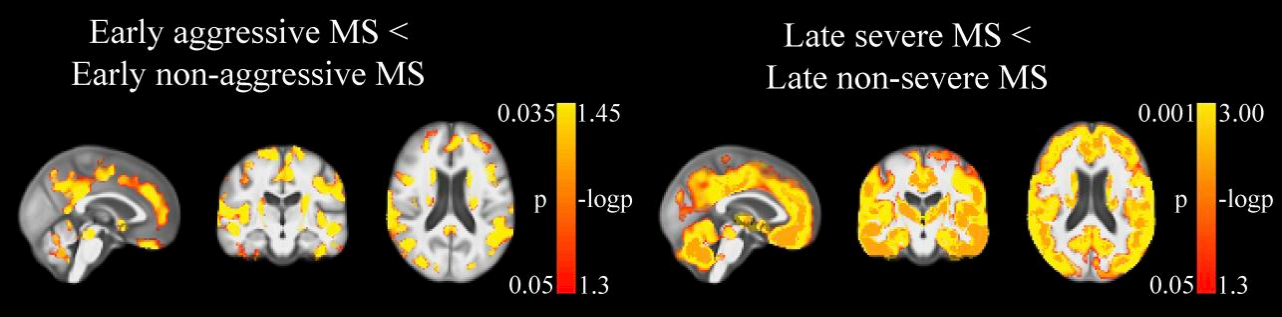
**Statistical analyses of voxel-based morphometry of the grey matter.** Permutation testing with threshold-free cluster enhancement was used while correcting for age and sex. False discovery rate *P* < 0.05 voxels shown. Significant clusters of decreased GM volume are overlaid on the study-specific template in which the mid-sagittal, -coronal and -ax Early aggressive multiple sclerosis and early non-aggressive multiple sclerosis groups had 86 individuals in each group. Late severe multiple sclerosis and late non-severe multiple sclerosis groups had 368 individuals in each group.

Tables 3 and 4 also show brain volume characteristics of the study groups. Early aggressive multiple sclerosis showed significantly lower TV (*P* < 0.001) and cortical volume (*P* = 0.035) and significantly higher LVV (*P* = 0.013), compared to early non-aggressive multiple sclerosis (Table 3). In VBM analysis, decreased GM volume was seen throughout the cortex as well as in the deep GM (Fig. 5); there were no areas where early non-aggressive multiple sclerosis had less GM volume. Early non-aggressive multiple sclerosis showed significantly lower whole brain volume, GMV, WM volume, TV (all *P* < 0.001) and cortical volume (*P* = 0.001) and significantly higher LVV (*P* < 0.001), compared to NHI.

Late severe multiple sclerosis showed significantly lower MUCCA, cortical volume, GMV, TV and whole brain volume and significantly higher LVV (all *P* < 0.001), compared to late non-severe multiple sclerosis (Table 4). In VBM analysis, decreased GM volume was seen throughout the entire cortical ribbon as well as deep GM (Fig. 5). Late severe multiple sclerosis did not present with any areas of significantly increased or decreased GM volume compared to their early aggressive counterparts. Late non-severe multiple sclerosis showed significantly lower whole brain volume, GMV, WM volume, TV (all *P* < 0.001) and cortical volume (*P* = 0.008) and significantly higher LVV (*P* < 0.001), compared to NHI.

### MRI differentiators of early aggressive and non-aggressive multiple sclerosis and late severe and non-severe multiple sclerosis

In a logistic stepwise regression model, early non-aggressive multiple sclerosis was differentiated from early aggressive multiple sclerosis by lower T2-LV (*B* = −0.638, SE = 0.240, Wald = 7.032, OR = 0.529, 95% CI [0.330, 0.847], *P* = 0.008) and higher TV (*B* = 0.603, SE = 0.238, Wald = 6.434, OR = 1.828, 95% CI [1.147, 2.913], *P* = 0.011). Goodness-of-fit tests (Hosmer-Lemeshow) indicated acceptable fit: χ² = 6.024, *P* = 0.645. The model explained 21.8% (Nagelkerke *R*^2^) of the variance in the disease group. The final model correctly classified 70.9% of cases.

In a logistic stepwise regression model, non-late severe multiple sclerosis was best differentiated from late severe multiple sclerosis by higher MUCCA (*B* = 0.872, SE = 0.161, Wald = 29.294, OR = 2.391, 95% CI [1.744, 3.279], *P* < 0.001) and cortical GMV (*B* = 0.604, SE = 0.171, Wald = 12.489, OR = 1.829, 95% CI [1.309, 2.556], *P* < 0.001). Model fit was adequate per the Hosmer-Lemeshow test: χ² = 9.240, *P* = 0.322. The model explained 24.8% (Nagelkerke *R*²) of the variance in disease group. The final model correctly classified 70.3% of cases.

### Linear regression modelling of severity of disability in early aggressive and late severe multiple sclerosis

In early aggressive multiple sclerosis, higher EDSS was predicted only by higher cortical LV (*B* = 0.167, 95% CI [0.021, 0.313], SE = 0.073, *t* = 2.287, *P* = 0.025. The model explained 24.9% of the variance in EDSS (adjusted *R*² = 0.180), *P* = 0.004.

In late severe multiple sclerosis, higher EDSS was significantly predicted by lower MUCCA (*B* = −0.252, 95% CI [−0.390, −0.115], SE = 0.069, *t* = −3.635, *P* < 0.001), higher cortical LN (*B* = 0.191, 95% CI [0.055, 0.328], SE = 0.069, *P* = 0.006) and lower TV (*B* = −0.153, 95% CI [−0.285, −0.021], SE = 0.067, *t* = −2.299, *P* = 0.024). The model explained 42.2% of the variance in EDSS (adjusted *R*² = 0.374), *P* < 0.001.

## Discussion

Severe multiple sclerosis is a disease form marked by rapid or delayed severe disability progression, which can follow distinct temporal trajectories (early and late).^4-7^ This retrospective study used demographic, clinical and MRI data collected over a period of 16 years, allowing a cross-sectional analysis with temporally distinct subgroups.

Compared to the literature, we found a similar proportion of early aggressive multiple sclerosis (4.4%),^4,8,12^ but a somewhat higher proportion of late severe multiple sclerosis (21.8%),^4,6^ in the BUFFALO-MS database.^24,26,27^ This can be attributed to ageing demographics and advancements in therapies delaying the onset of severe multiple sclerosis in later age decades. There is a need for more attention on studying severe multiple sclerosis cases due to their pronounced clinical burden, complexity and high prevalence (>25% of our cohort).

Compared to matched multiple sclerosis controls, early aggressive multiple sclerosis stood out for its association with the presence of higher T2 hyperintense, T1 hypointense and cortical lesion burden early in the disease, in addition to more advanced thalamic, cortical and central atrophy and higher degree of thalamic dysconnectivity. In logistic regression analysis, thalamic atrophy and higher T2 hyperintense lesion burden were the only differentiators between early aggressive and non-aggressive multiple sclerosis. Our findings support results from prior studies identifying thalamic atrophy,^21,51,52^ thalamic dysconnectivity^40^ and WM lesion burden^17^ as critical drivers of disability progression in early multiple sclerosis. Higher cortical lesion burden was the only significant predictor of disability progression in regression analyses in early aggressive multiple sclerosis in this study. These results were corroborated by LPM and VBM findings, indicating that the presence of eloquent lesions (T2-LV and T1-LV) in specific CNS areas, as well as advanced cortical and thalamic atrophy are key features of aggressive multiple sclerosis. A recent study showed a key role of GM atrophy (cortical and thalamic) in severe multiple sclerosis,^6^ further highlighting the need to determine the extent of GM pathology in early aggressive multiple sclerosis^20^ from the earliest clinical stages. We did not find that SC lesion burden or atrophy was a significant MRI differentiator between early aggressive and non-aggressive multiple sclerosis or that explained additional variance when predicting disability, implying more widespread supratentorial pathology. However, we cannot exclude that SC damage plays an important role in early disability accrual, as 25% of the study sample lacked MRI SC examination. Nevertheless, our data suggest that cerebral GM damage (thalamus and cortex) may play a more important role than SC damage for advancing disability progression in the early stages of multiple sclerosis and therefore studying cognitive function in future studies of this disease phenotype may be relevant.

Early aggressive multiple sclerosis demonstrated unique demographic patterns, with a notably higher prevalence of African-Americans and males compared to late severe multiple sclerosis. African-Americans with multiple sclerosis have been shown to have higher disability rates and faster progression, which could contribute to the disproportionate representation in early aggressive multiple sclerosis.^53^ These demographic trends may point to genetic, environmental or socio-economic factors influencing disease severity and progression, warranting further investigation into tailored therapeutic approaches for these populations.^1^ In addition, we found significantly higher age at onset in early aggressive compared to late severe multiple sclerosis. Studies have shown that older age at onset correlates with a higher risk of aggressive multiple sclerosis, including rapid disability accumulation, reduced relapse-free periods and earlier onset of progressive multiple sclerosis.^1,9,13,14^

Conversely, late severe multiple sclerosis exhibited disability progression strongly linked to SC and cortical lesion burden and atrophy, thalamic damage and global brain volume loss and lesion burden accumulation. In logistic regression analysis, lower MUCCA and cortical GM volume were the best differentiators between late severe and non-severe multiple sclerosis. In linear regression analyses, lower MUCCA and TV, along with greater cortical lesion burden, best explained disease severity, as reflected by the EDSS. Like in early aggressive multiple sclerosis, LPM and VBM findings in late severe multiple sclerosis identified a cluster in the entire WM and increased cortical LV probability was seen throughout much of the cortex, while the VBM analysis showed decreased GM volume throughout the entire cortical ribbon as well as in deep GM. These findings suggest that GM atrophy in early aggressive multiple sclerosis is related to primary GM damage, whereas the one in the late severe multiple sclerosis is more combination of the primary and secondary (WM lesions leading to secondary retrograde Wallerian degeneration) GM damage, indicating insidious and slower degenerative global disease processes. These results underscore the heterogeneity of severe multiple sclerosis and the importance of distinguishing between early and late severe multiple sclerosis for targeted diagnosis, monitoring and treatment. In late severe multiple sclerosis, the prominence of SC damage parallels findings by a recent study emphasizing the prognostic importance of SC pathology for the development of progressive multiple sclerosis.^19^ Age-related changes, comorbidities and a slower accrual of damage may amplify the effects of global CNS pathology, contributing to the distinct imaging trajectory of late severe compared to early aggressive multiple sclerosis.^1,3^ The cumulative burden of neurodegeneration and reduced neuroplasticity in older individuals further differentiates late severe multiple sclerosis from its early aggressive counterpart.

Recent advancements in imaging techniques and AI-based methods provide opportunities to utilize legacy datasets for retrospective cortical lesion burden assessment. Methods like FLAIR2,^30^ T1/T2 ratio,^32^ AI-double inversion recovery^31^ and multi-modal cortical lesion enhanced enable the extraction of additional latent information from conventionally acquired imaging data, offering enhanced sensitivity to cortical lesion contrast. By applying these techniques, retrospective quantifications can be performed on existing datasets without requiring new acquisitions. This is one of the first studies in multiple sclerosis, to show that application of AI-based algorithms further allows analysis and precise quantification of cortical lesion burden on MRI scans acquired over a large period. This opens possibilities for reanalysing large-scale legacy clinical datasets to derive novel insights into monitoring disease progression and treatment efficacy, as shown in this study.

The assessment of chronic active lesions is important, as they signify extensive demyelination and microglia activation, correlating strongly with disease progression, and provide deeper insights into multiple sclerosis pathology.^22-24^ Because these lesions highlight ongoing inflammation and neurodegeneration, offering insights into the timing and mechanisms of therapeutic intervention to prevent irreversible damage, it would be important to study the incidence and prevalence of chronic active lesions in early aggressive and late severe multiple sclerosis. In this study, we did not assess paramagnetic rim lesions, as the susceptibility-WI sequence for the determination of these lesions was not available throughout the entire period of data collection, which should be done in future studies.

The findings from this study have implications for clinical practice, particularly in the early identification and management of severe multiple sclerosis. In early aggressive multiple sclerosis, the strong association between cortical lesions, WM lesion burden, thalamic damage and disability progression underscores the need for advanced imaging protocols focusing on assessing such pathologies in clinical routine and use of potent anti-inflammatory and neuroprotective therapies to slow down the disease progression. Identifying African-Americans and males as higher-risk groups further suggests that these populations should be prioritized for early and aggressive intervention. In late severe multiple sclerosis, SC imaging should be included in disease monitoring, given the critical role of SC damage in association with disability. Assessment of global-, regional- and tissue-specific brain atrophy is also important for monitoring and predicting disease progression, offering additional targets for therapeutic intervention.^1^ Therefore, therapies tailored to the prevention of microglia activation, CNS atrophy and GM damage should be prioritized to manage the unique challenges posed by severe multiple sclerosis and improve patient outcomes.^1^

Previous research investigated multiple sclerosis phenotypes based on rapid disability accrual and early brain atrophy.^51,52^ Additionally, the MAGNIMS consortium has emphasized the prognostic value of early MRI metrics.^51,52^ However, our study is among the first to use a large, longitudinal real-world database with >15 years of standardized imaging and to directly compare early aggressive and late severe multiple sclerosis trajectories using detailed neuroimaging phenotypes, including SC, cortical and thalamic connectivity measures. The clinical utility of our findings lies in refining phenotypic stratification for future prognostic modelling and in informing patient selection for early aggressive and late severe treatment strategies.

This study’s retrospective design introduces inherent limitations, including potential selection biases and variability in MRI protocols over time. Although all subjects in the BUFFALO-MS database were reclassified in 2024 according to the 2017 McDonald criteria, we acknowledge that the cohort spans different diagnostic eras, which may have introduced heterogeneity in disease severity at the time of the MRI examination. All MRI scans were acquired on the same 1.5T and 3T GE scanners (used consistently from 2007 to the present), with only one software upgrade implemented in 2017. Nevertheless, differences in acquisition protocols before and after the upgrade may have influenced the sensitivity to detect certain MRI abnormalities. Additionally, patients with longer follow-up may inherently represent those with more advanced disease stages, potentially biasing associations towards greater clinical progression while paradoxically reducing sensitivity for imaging markers due to older and lower-resolution scans. While propensity matching was employed to control for confounders, the cross-sectional nature of the data prevents conclusions about causality or longitudinal changes. Additionally, the generalizability of the findings may be constrained by the demographic composition of the study population, which primarily consisted of patients from a single geographic region, so future studies should validate these results across different populations.

In conclusion, we characterized neuroimaging phenotypes of early aggressive and late severe multiple sclerosis, highlighting the critical role of cortical lesions, WM lesion burden and thalamic pathology in early aggressive multiple sclerosis and SC, cortical, thalamic and widespread pathology in late severe multiple sclerosis. Early and late severe multiple sclerosis have distinct initial demographic, clinical and neuroimaging phenotypes but share common GM-based neurodegeneration.

## Supplementary Material

fcaf254_Supplementary_Data

## Data Availability

The data that support the findings of this study are available from the corresponding author upon reasonable request. Data sharing is not applicable to this article as no new data were created or analysed in this study. No custom code was developed for this work.
