# Supplementary material for: Neuroimaging phenotype characterization of early aggressive and late severe multiple sclerosis: a case-control study
Source: Brain Commun. 2025 Jun 24;7(4):fcaf254. doi: 10.1093/braincomms/fcaf254 (PMC12238752; doi:10.1093/braincomms/fcaf254)
Supplement: fcaf254_Supplementary_Data [file fcaf254_supplementary_data.docx]

**Supplementary Table 1**. Brain and cervical spinal cord acquisition protocols in the study population.

|  | Pre-software upgrade | | Post-software upgrade | |
| --- | --- | --- | --- | --- |
|  | 1.5 T | 3T | 1.5T | 3T |
| **Brain MRI** | | | | |
| SE T2-FLAIR  (Plane/Mode/ TE/TI/TR/ voxel size) | Axial/2D/ 120/2000/8000 1.0x1.0x3.0 mm^3^ | Axial/2D/ 120/2100/8500 1.0x1.0x3.0 mm^3^ | Axial/3D/ 133/1848/6000/ 1.0x1.0x1.2mm^3^ | Axial/3D/ 104/2407/9000/ 1.0x1.0x1.2mm^3^ |
| GRE T1  (Plane/Mode/ TE/TI/TR/ voxel size) | Axial/3D/ 3.7/900/7.7/ 1.0x1.0x1.0mm^3^ | Axial/3D/ 2.8/900/5.9/ 1.0x1.0x1.0mm^3^ | Sagittal/3D/ 3.9/500/9.0/ 1.0x1.0x1.2mm^3^ | Sagittal/3D/ 2.2/600/5.4/ 1.0x1.0x1.2mm^3^ |
| SE T1  (Plane/Mode/ TE/TR/ voxel size) with and without use of 0.1 mMol/kg of gadolinium contrast | Axial/2D/ 12/450/ 0.9x0.9x1mm^3^ | Axial/2D/ 15/600/ 1.0x1.0x3.0mm^3^ | Sagittal/3D/ 10.3/550/ 1.0x1.0x1.0mm^3^ | Sagittal/3D/ 14/533/ 1.0x1.0x0.9mm^3^ |
| Dual echo SE T2/PD  (Plane/Mode/ TE1/TE2/TR/ voxel size) | Axial/2D/ 10/89/7467 0.9x0.9x1mm^3^ | Axial/2D/ 10/90/3000 1.0x1.0x1.0mm^3^ | N/A | N/A |
| SE T2  (Plane/Mode/ TE/TR/ voxel size) | N/A | N/A | Axial/2D/  98/3600/ 0.9x0.9x3.0mm^3^ | Axial/2D/ 104/4350/ 0.9x0.9x3.0mm^3^ |
|  | | | | |
| **Cervical spinal cord MRI** | | | | |
| T2-STIR  (Plane/Mode/ TE/TI/TR/ voxel size) | Sagittal/2D/ 31/150/5517 1.0x1.0x3.0mm^3^ | Sagittal/2D/ 41/190/4300/ 0.9x0.9x2.0mm^3^ | Sagittal/2D/ 40/150/3500/ 1.3x1.3x3.0mm^3^ | Sagittal/2D/ 49/190/5050/ 0.7x0.7x3.0mm^3^ |

**Legend:** SE – spin echo; FLAIR – fluid attenuated inversion recovery; TE – echo time; TI – inversion time; TR – repetition time; GRE – gradient echo; PD – proton density;
N/A – not applicable; STIR – short tau inversion recovery.

The software on both scanners was upgraded in November 2017 (from version 12\LX\MR Software release:12.0_M5_0606.b to 23\LX\MR Software release:HD23.0_V03_1614.b).
